# Coupling of Lever Arm Swing and Biased Brownian Motion in Actomyosin

**DOI:** 10.1371/journal.pcbi.1003552

**Published:** 2014-04-24

**Authors:** Qing-Miao Nie, Akio Togashi, Takeshi N. Sasaki, Mitsunori Takano, Masaki Sasai, Tomoki P. Terada

**Affiliations:** 1 Department of Computational Science and Engineering, Nagoya University, Nagoya, Japan; 2 Institute for Molecular Science, Okazaki, Japan; 3 Department of Applied Physics, Zhejiang University of Technology, Hangzhou, P. R. China; 4 Department of Human Informatics, Aichi Shukutoku University, Aichi, Japan; 5 Department of Physics, Waseda University, Ohkubo, Shinjuku-ku, Tokyo, Japan; 6 School of Computational Sciences, Korea Institute for Advanced Study, Seoul, Korea; University of California, Santa Barbara, United States of America

## Abstract

An important unresolved problem associated with actomyosin motors is the role of Brownian motion in the process of force generation. On the basis of structural observations of myosins and actins, the widely held lever-arm hypothesis has been proposed, in which proteins are assumed to show sequential structural changes among observed and hypothesized structures to exert mechanical force. An alternative hypothesis, the Brownian motion hypothesis, has been supported by single-molecule experiments and emphasizes more on the roles of fluctuating protein movement. In this study, we address the long-standing controversy between the lever-arm hypothesis and the Brownian motion hypothesis through *in silico* observations of an actomyosin system. We study a system composed of myosin II and actin filament by calculating free-energy landscapes of actin-myosin interactions using the molecular dynamics method and by simulating transitions among dynamically changing free-energy landscapes using the Monte Carlo method. The results obtained by this combined multi-scale calculation show that myosin with inorganic phosphate (P_i_) and ADP weakly binds to actin and that after releasing P_i_ and ADP, myosin moves along the actin filament toward the strong-binding site by exhibiting the biased Brownian motion, a behavior consistent with the observed single-molecular behavior of myosin. Conformational flexibility of loops at the actin-interface of myosin and the N-terminus of actin subunit is necessary for the distinct bias in the Brownian motion. Both the 5.5–11 nm displacement due to the biased Brownian motion and the 3–5 nm displacement due to lever-arm swing contribute to the net displacement of myosin. The calculated results further suggest that the recovery stroke of the lever arm plays an important role in enhancing the displacement of myosin through multiple cycles of ATP hydrolysis, suggesting a unified movement mechanism for various members of the myosin family.

## Author Summary

Myosin II is a molecular motor that is fueled by ATP hydrolysis and generates mechanical force by interacting with actin filament. Comparison among various myosin structures obtained by X-ray and electron microscope analyses has led to the hypothesis that structural change of myosin in ATP hydrolysis cycle is the driving mechanism of force generation. However, single-molecule experiments have suggested an alternative mechanism in which myosin moves stochastically in a biased direction along actin filament. Computer simulation serves as a platform for assessing these hypotheses by revealing the prominent features of the dynamically changing landscape of actin-myosin interaction. The calculated results show that myosin binds to actin at different locations of actin filament in the weak- and strong-binding states and that the free energy has a global gradient from the weak-binding site to the strong-binding site. Myosin relaxing into the strong-binding state therefore necessarily shows the biased Brownian motion toward the strong-binding site. Lever-arm swing is induced during this relaxation process; therefore, lever-arm swing and the biased Brownian motion are coupled to contribute to the net displacement of myosin. This coupling should affect the dynamical behaviors of muscle and cardiac systems.

## Introduction

Myosin II, the conventional myosin responsible for muscle contraction, generates mechanical force by interacting with actin filament. Our understanding of this actomyosin motor has greatly increased by X-ray analyses of myosin structures [1]–[3] and by electron microscopy (EM) of actomyosin complex [4]–[7]. These structural observations have led to the widely held lever-arm hypothesis [2], [3], in which the change in the nucleotide state in the myosin head is amplified through allosteric communication for rotating the lever-arm region of myosin to exert mechanical force. X-ray and EM data of static protein structures do not, however, provide direct information on how the motor works dynamically. Dynamical behaviors have been observed in single-molecule experiments (SMEs) [8]–[14], among which the Yanagida group [11], [13] analyzed the fluctuating motion of a single subfragment-1 (S1) of myosin and supported the alternative Brownian-motion hypothesis [15]. In this hypothesis, the myosin head stochastically moves along the actin filament with a regular step size of 5.5 nm, which corresponds to the diameter of actin subunit, in both directions toward the plus and minus ends of the actin filament during a single cycle of ATP hydrolysis. In this stochastic walk or effective Brownian motion, the frequency of steps toward the plus end is considerably higher than that of steps to the minus end. This biased Brownian motion enables the search for a stable binding site on the filament, which pulls the filament to exert mechanical force [11], [13].

The thermal Brownian fluctuation of the myosin molecule should also cause the stochastic fluctuation in the direction of lever-arm swing. Even with such Brownian fluctuation of conformation, the lever-arm hypothesis implies that the net displacement of myosin is limited by the allowed angular range of the lever arm. In contrast to this narrow distribution, the net displacement of myosin stochastically varies under the Brownian-motion hypothesis and its distribution is broad and changes flexibly depending on the load applied to the system. These two hypotheses should accordingly show a clear difference in predicting the flexibility and load dependence of the system [16]. In addition, for myosin V, a non-conventional myosin responsible for vesicle transport, SME measurements [17]–[19] have clearly shown that the Brownian motion of the leading head of myosin in searching for the binding location on the actin filament significantly contributes to force generation together with the lever-arm pushing mechanism at the trailing head of myosin. A key issue in understanding the mechanism of actomyosin motors is thus to clarify how and to what extent lever-arm swing and Brownian motion contribute to force generation [16], [20], [21]. In this study, we address this problem by *in silico* observations of the system composed of a single head (S1) of myosin II and an actin filament.

Analyses of the kinetic cycle of interactions between myosin II and actin filament [22] should help to resolve this problem:
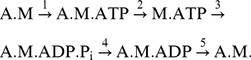
(1)Myosin (M) strongly binds to actin filament (A) when no nucleotide is bound to myosin to form the rigor state (A.M). When ATP binds to myosin (A.M.ATP), the myosin detaches from the actin filament (M.ATP). After the bound ATP is hydrolyzed into ADP and P_i_ , the complex M.ADP.P_i_ binds to actin to form the weakly bound state (A.M.ADP.P_i_), which is transformed to the strongly bound state by the release of P_i_ (A.M.ADP) and ADP to reach the rigor state again. From observed structures of myosin with various nucleotide analogs [2], [3], [23], it is plausible to assume that the lever arm of myosin in M.ATP and M.ADP.P_i_ is in the pre-stroke position and the lever arm in other states is in the post-stroke position; therefore, processes 4 and 2 in Eq.1 should correspond to lever-arm stroke during force generation and the recovery stroke, respectively.

**Figure 1:**
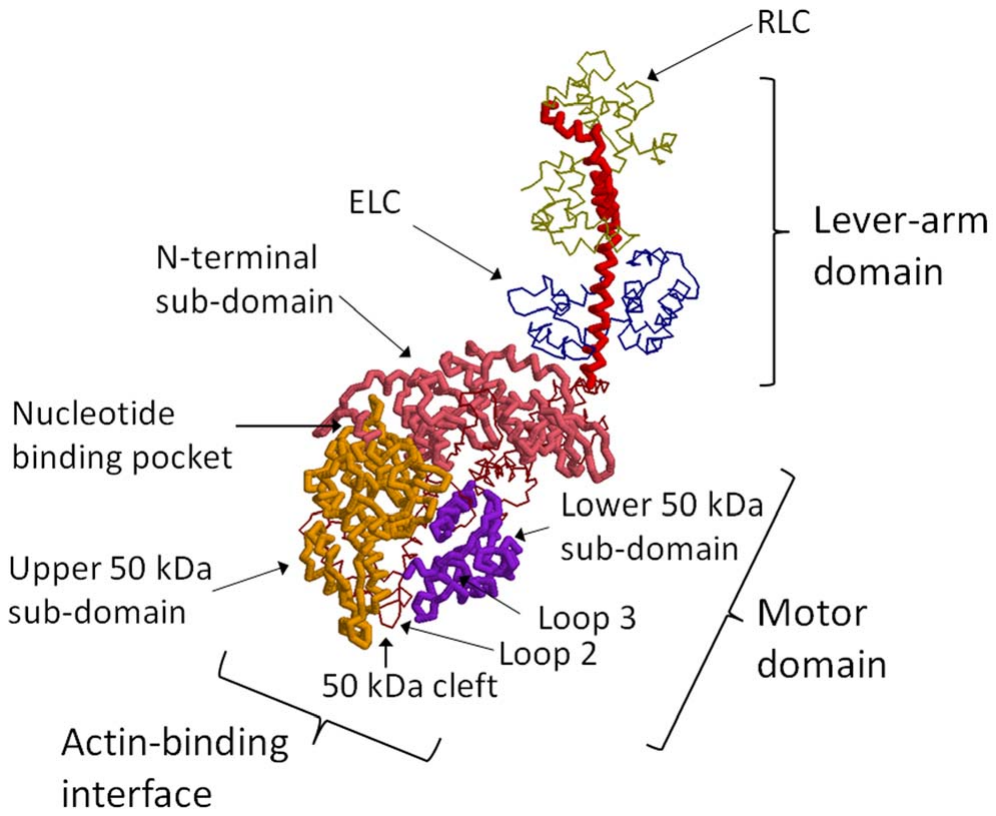
Structure of S1 of myosin II obtained by fitting the EM data[7]. S1 comprises a heavy chain, an essential light chain (ELC, blue thin line), and a regulatory light chain (RLC, dark yellow thin line). The heavy chain is composed of the lever-arm domain (red thick line) and the motor domain. In the motor domain, the N-terminal subdomain (pink), the upper 50 kDa subdomain (orange), and the lower 50 kDa subdomain (purple) are drawn with thick lines. The 50 kDa cleft is the interspace between the upper and lower 50 kDa subdomains. The nucleotide binding pocket lies between the upper 50 kDa subdomain and the N-terminal subdomain. The actin-binding interface of myosin includes the region around the 50 kDa cleft, loop 2, loop 3, and other loops.

Further detailed comparison among kinetic states and structures, however, has raised a question regarding the application of the lever-arm hypothesis [3]. From various observed myosin structures, it is noted that the opening/closure of the nucleotide binding pocket, the lever-arm positioning, and the closure/opening of the 50 kDa cleft of myosin are correlated with one another [23], [24] (See Fig. 1 for an example structure of S1 of myosin II). The resolved structures have shown that P_i_ in M.ADP.P_i_ makes the nucleotide binding pocket closed, which tends to maintain the lever-arm in the pre-stroke position and the 50 kDa cleft open. Given that the closure of the 50 kDa cleft has been reported to be necessary for the strong binding of myosin to actin [4]–[7], it is reasonable to assume that M.ADP.P_i_ weakly binds to actin. The weak binding of M.ADP.P_i_ to actin has been suggested by kinetic [25]–[27] and structural [28]–[30] measurements. However, for myosin to exert a force using the lever-arm mechanism, myosin must strongly bind to actin before the occurrence of the lever-arm swing. This problem in applying the lever-arm hypothesis may be solved if it is assumed that the 50 kDa cleft of A.M.ADP.P_i_ is closed, although the pre-stroke open-cleft structure is stable in M.ADP.P_i_ [31]. If myosin adopts the pre-stroke closed-cleft structure, it should strongly bind to the actin filament, and the subsequent occurrence of the lever-arm swing on the release of P_i_ should generate mechanical force. The pre-stroke closed-cleft structure may be possible when this structure is stabilized by specific myosin-actin interactions. Although considerable effort has been devoted to detecting the pre-stroke closed-cleft structure [32], there is no direct evidence for its existence thus far [33].

In this study, we develop a theory on kinetic process, which is a dynamical energy landscape theory of actomyosin, without relying on the assumption of a stable pre-stroke closed-cleft structure of myosin. We assume that myosin interacting with actin tends to adopt one of structures observed in previous experiments. We also assume that the structure of myosin with a given nucleotide-binding state shows fluctuating transitions among these conformations, such as are shown by many allosteric proteins in the population-shift or conformation-selection mechanism of allostery [34], [35].

In our previous studies, the theoretical models of movement of myosin S1 were discussed [36], [37]. Molecular dynamics simulation was performed to investigate myosin with the nucleotide-free post-stroke closed-cleft structure [37] and it was shown that the electrostatic interactions at the actin-myosin interface should lead to a globally biased energy landscape of myosin movement toward the strong-binding site on the actin filament and that the stochastic movement of weakly binding myosin follows the gradient of this landscape in the course of relaxation from weak- to strong-binding states; therefore, the relaxation process reproduces the biased Brownian motion observed in SMEs [11], [13]. However, to investigate the roles of this simulated behavior in the kinetic cycle of Eq.1, as noted in [37], we need to extend this method to cases in which the energy landscape is not fixed, but is dynamically changing according to changes in nucleotide state and conformation.

**Figure 2:**
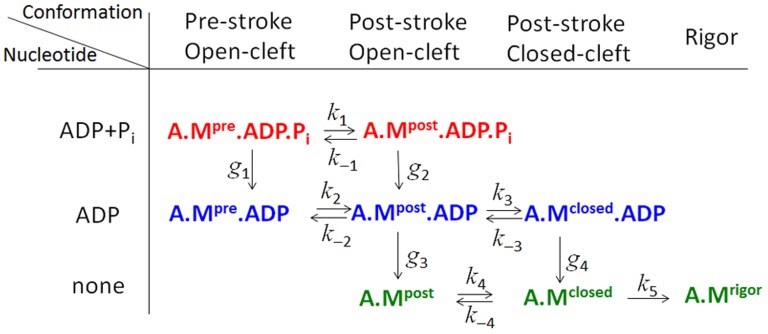
The kinetic network among actomyosin states considered in the present simulation. Each state is defined by the nucleotide state and the myosin conformation, and 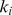 and 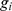 are rates of transitions between actomyosin states.

In dynamical energy landscape theory, multiple kinetic states, corresponding to different stages of chemical reactions or other different conditions, are considered and the dynamical switching among landscapes in these states is analyzed [38]–[45]. Here we consider the multiple kinetic states appearing in the course of force generation, called “actomyosin states.” Figure 2 shows the kinetic network among actomyosin states considered in this study. Actomyosin states shown in Fig. 2 are defined by both the conformation and nucleotide state of myosin. We assume that myosin in actomyosin states tends to adopt conformations similar to those observed in X-ray or EM data. Myosin in A.M.ADP.P_i_ should adopt the pre-stroke open-cleft conformation (M^pre^) that is modeled by the X-ray structure of myosin with an ADP.P_i_ analog, and myosin in A.M.ADP should adopt the post-stroke open-cleft conformation in the X-ray data (M^post^). Myosin in A.M should adopt the post-stroke closed-cleft conformation (M^closed^) obtained by fitting the EM image in the rigor state. See the *Methods* section for more details on the definitions of these model conformations. In the present study, we distinguish the weakly bound M^closed^ from the strongly bound M^rigor^ in the rigor state. Although both M^closed^ and M^rigor^ have a post-stroke closed-cleft conformation, water molecules that hydrate myosin should be expelled from the interface with actin in transition to the rigor state, which is expressed in the model by a transition from M^closed^ to M^rigor^.

It should be noted that in A.M.ADP in the absence of P_i_, switch-I and switch-II regions of myosin are not bound to the ligand, and therefore, the post-stroke position of the lever arm and the closed 50 kDa cleft are expected to be energetically stable. The open 50 kDa cleft structure, however, should be entropically favorable to form both the post-stroke open-cleft structure and post-stroke closed-cleft structure in A.M.ADP. Therefore, we consider that A.M.ADP fluctuates between A.M^post^.ADP and A.M^closed^.ADP. Though the post-stroke open-cleft structure M^post^ has been often referred to as the “near-rigor” or “post-rigor” conformation that appears after leaving the rigor state [46], the post-stroke open-cleft structure is a representative structure of the ADP-bound myosins, and there is no evidence against the appearance of this structure before the rigor state is reached. Therefore, we use M^post^ as a structure expected in A.M.ADP. Similarly, we consider that both M^post^ and M^closed^ appear in A.M. The electron paramagnetic resonance data have shown that coupling between the nucleotide state and conformation is not rigid [47]. We, therefore, assume that myosin with a given nucleotide state can adopt conformations that are expected to appear in the next or in the previous step of ATP hydrolysis as pre-existing or post-existing conformations in the conformation-selection mechanism of allostery. We consider A.M^post^.ADP.P_i_ to be the pre-existing conformation (the conformation expected to be found in the ADP bound state). In the ADP-bound state, we consider A.M^pre^.ADP to be the post-existing conformation (the conformation expected in the ADP.P_i_ bound state). The rigor state is reached through the conformation-selection mechanism by selecting the pre-existing M^closed^ conformation in the weakly bound state. It is assumed that the concentration of ADP or P_i_ in solution is so low that reverse reactions in steps of ATP hydrolysis are negligible. Thus, we have the network of transitions as shown in Fig. 2. We also assume that the strongly bound state A.M^rigor^ is stable, and hence, we do not consider the spontaneous loosening of binding from A.M^rigor^ to A.M^closed^.

**Figure 3:**
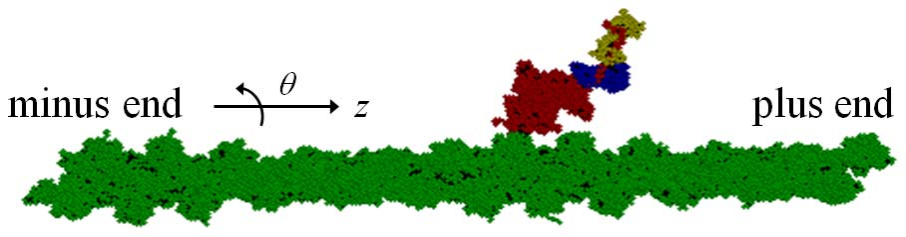
The setup for the Langevin molecular dynamics simulations of actomyosin. An S1 of myosin II (red) with an essential light chain (ELC, blue) and a regulatory light chain (RLC, yellow) is placed on an actin filament (green), which lies along the 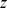 direction. 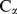
s of the actin filament are connected to spatially fixed points by springs, and the lever-arm tip of myosin is constrained to move along a line that runs parallel to the actin filament. The angle around the 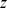-axis is denoted by 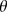.

For individual actomyosin states, we calculate the free-energy landscape which determines the movement of the myosin head in each of these states. Free-energy landscapes of myosin movement and actin-myosin binding are derived using a coarse-grained model of actomyosin, which represents proteins as chains connecting beads of 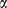 carbons (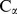
s). Forces acting among 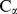
s of myosin are derived from the Gō-like potential [48], [49], which stabilizes the model myosin structure, M^pre^, M^post^ or M^closed^. Nucleotide and Mg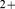 ion bound to myosin are represented as particles of all nonhydrogen atoms. In this way, different actomyosin states are represented using different Gō-like potentials and different models of nucleotide and Mg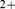. The potential consistently used among actomyosin states is the Gō-like potential for actin, which stabilizes the EM structure of actin filament [50]. As inter-protein interactions, we introduce electrostatic interactions, which are represented by Debye-Hückel potentials, and van der Waals interactions, which are represented by the Lennard-Jones type potentials. Using these potentials, we perform the Langevin molecular dynamics simulation. The setup of the simulation is shown in Fig. 3. An S1 domain of myosin II, comprising a heavy chain, an essential light chain (ELC), and a regulatory light chain (RLC), is placed on the actin filament, which extends along the 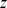-axis with its plus-end facing the positive 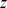 direction. The angle around the 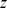-axis is denoted by 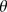. The actin filament is connected to the spatially fixed points by springs. By mimicking the setup of the SME [11], the tip of the myosin lever-arm is connected by springs to a line running parallel to the actin filament. Myosin can move freely along this line without any bias either toward the 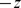 or 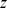 direction. By monitoring the position 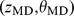 of the center of mass of the myosin motor domain (MD) during simulations, we calculate the free-energy landscape in the two-dimensional space of 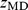 and 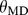 using the weighted histogram analysis method (WHAM) [51] with umbrella potentials. See the *Methods* section for the simulation details.

Using the free-energy landscapes thus calculated, the movement of myosin II on the surface of an actin filament is simulated by the stochastic motion of a point at the center of mass of the myosin motor domain. Motion of this point along the free-energy landscape of each state is simulated using the value of the free energy in the Metropolis algorithm. The transition between different actomyosin states because of nucleotide-state change or lever-arm swing is simulated by dynamical switching between free-energy landscapes. Therefore, the point representing the position of myosin moves along the calculated free-energy landscapes and stochastically jumps among them. In this way, we shed light on roles of both the lever-arm swing, occurring during transitions among landscapes, and the biased Brownian motion along individual landscapes.

## Results

### Free-energy landscapes in actomyosin states

**Figure 4:**
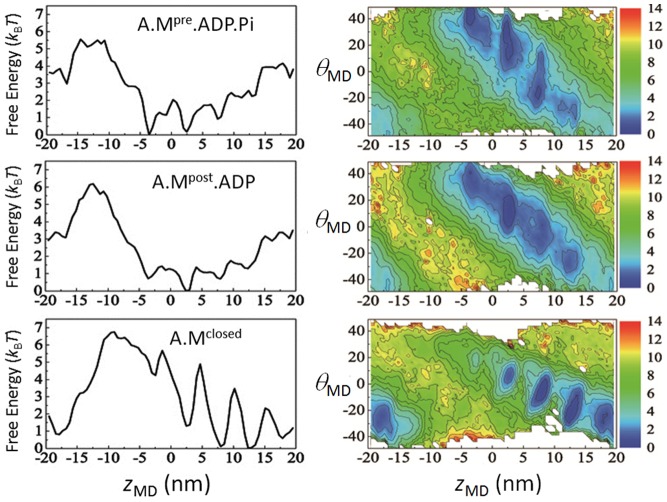
Free-energy landscapes of actin-myosin interactions. Two-dimensional free-energy landscapes are drawn in the plane of the coordinate of the center of mass of the myosin head 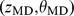 as contour maps in units of 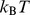 (right), and one-dimensional free-energy landscapes on the coordinate 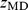 (left). Landscapes in A. M^pre^.ADP.P_i_ (top), A.M^post^.ADP (middle), and A.M^closed^ (bottom).

In Fig. 4, free-energy landscapes of actin-myosin interaction in states A.M^pre^.ADP.P_i_ , A.M^post^.ADP, and A.M^closed^ are shown as functions of 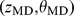. In addition, the one-dimensional free-energy landscapes obtained by projecting the two-dimensional landscapes onto the 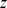-axis are shown. The calculated free-energy landscapes are almost periodic in the 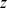 direction because of the helical nature of the EM structure of the actin filament with approximate helical pitch 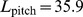 nm. In states with the open 50 kDa cleft structure, A. M^pre^.ADP.P_i_ (top, Fig. 4) and A.M^post^.ADP (middle, Fig. 4), the landscapes have multiple basins located at an interval of 5.5 nm, corresponding to the diameter of the actin subunit. These basins are separated by the low free energy barrier of 1–2 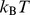, which should be easily overcome by thermal noise. The lowest free-energy minima on the landscape of A.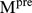.ADP.P_i_ and A.M^post^.ADP are positioned at 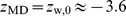 nm and 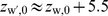 nm, respectively, as shown in Fig. 4.

A large difference from the above two landscapes is found in the landscape of the A.M^closed^ state with a closed 50 kDa cleft structure (bottom, Fig. 4). The landscape has an array of basins at positions separated by the size of actin subunit, 5.5 nm, with a global gradient toward the strong binding site at 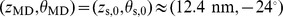. This prominent feature can be ascribed to complementary matching between the closed-cleft structure of myosin and the actin filament with a heterogeneous distribution of electric charges on its surface. The shear motion between upper and lower 50 kDa subdomains should also contribute to the complementary matching between myosin and actin in A.M^closed^ [52]. The arrangement of valleys in the 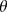 direction is also notable. In A. M^closed^ the angle difference between adjacent basins is considerably smaller than the angle expected from the helical structure of the filament, 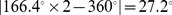. This narrow distribution of basins results from the interplay among the myosin-actin interactions and the restraints on the motion of myosin and actin. The disagreement between the actin-subunit arrangement and the basin distribution indicates that myosin binds with different orientations to the actin surface in different basins, a difference that should lead to the difference in free energy among these basins. Thus, the strong gradient of the free-energy landscape is coupled with a narrow distribution of basins in the landscape.

**Figure 5:**
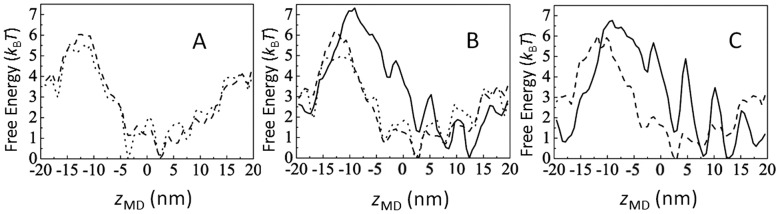
One-dimensional free-energy landscapes in different actomyosin states. Free-energy landscapes (A) in A.M.ADP.P_i_ with M being M^pre^ (dotted) and M^post^ (dashed), (B) in A.M.ADP with M being M^pre^ (dotted), M^post^ (dashed), and M^closed^ (solid), and (C) in A.M with M being M^post^ (dashed) and M^closed^ (solid).

The one-dimensional free-energy landscapes in seven states in Fig. 2 are compared in Fig. 5. It is found that the difference in conformation more significantly affects the free-energy landscape than the difference in the nucleotide state. The corresponding two-dimensional landscapes are shown in *Fig. S1*. We do not consider myosin movement in A.M^rigor^, and therefore, the calculation of free-energy landscape in the A.M^rigor^ state is omitted.

From Figs. 4 and 5, we can deduce the behavior of myosin through kinetic transitions in Fig. 2. A myosin head landing on the actin filament should be attracted to the valley in the free-energy landscape of A.M^pre^.ADP.P_i_. It should weakly bind there and widely fluctuate among multiple basins of landscapes in A.M^pre^.ADP.P_i_ or A.M^post^.ADP. The most populated 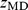-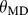 region of the myosin head in A.M^pre^.ADP.P_i_ , A.M^post^.ADP, or other states is the region of high free energy in A.M^closed^.ADP and A.M^closed^ landscapes. Thus, after releasing P_i_ or ADP.P_i_ , the myosin begins to relax to the more stable low free-energy position at the larger 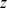 by moving along the actin filament. This movement associates jumps among minima with a regular spacing of approximately 5.5 nm.

### Diffusive motions and transitions

The above scenario of myosin movement can be verified by Monte Carlo (MC) simulation. The diffusive motion of the myosin head is simulated by the motion of a point representing the position of the center of mass of the myosin motor domain on the calculated two-dimensional free-energy landscape using the Metropolis algorithm. The trial movement of a point is generated as a step on the lattice with mesh size 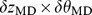, where 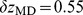 nm and 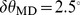. This trial is accepted when the free-energy change induced by the trial movement is 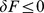. When 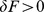, the trial is accepted with probability 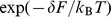 and rejected with probability 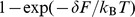. A similar method was used to simulate the movement of kinesin head along the surface of a microtubule [39].

We extend this method by applying it to the problem of multiple landscapes. A point representing the center of mass of the myosin motor domain diffuses along a landscape and jumps from 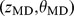 on one landscape to the same 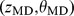 on the other landscape with probabilities defined by rates in Fig. 2; 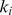 with 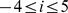 and 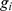 with 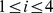. Values of 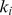 with 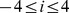 and 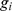 with 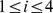 represent chemical reactions and large-scale conformational change, respectively, which should have a 1–10 ms timescale. As discussed in the *Methods*, 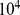 Monte Carlo steps (MCS) should correspond to several ms or longer, and hence, 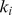 with 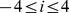 and 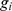 with 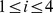 should be 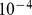
–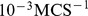. For simplicity, we use either of two values, 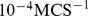 or 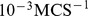. Given that M^pre^.ADP.P_i_, M^post^.ADP, and M^closed^ have been observed in the X-ray and EM analyses, the actomyosin states A.M^pre^.ADP.P_i_, A.M^post^.ADP, and A.M^closed^ should be relatively stable. In the following, values of 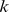 and 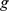 are chosen to stabilize the A.M^pre^.ADP.P_i_ state as 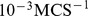 for 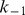, 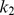, 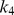, 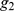, 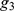 and 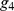, and 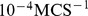 for 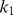, 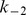, 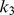, 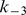, 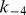 and 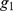. 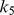 is the rate of the process of hydrophobic matching between surfaces of proteins and should be faster than the large conformational change of proteins. We accordingly use the value 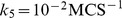. The calculated results are robust against changes in this parametrization. See *Fig. S2* for the results of other choices of values for 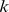 and 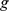.

After the transition from one landscape to the other, the point representing the center of mass of the myosin motor-domain continues to diffuse on the new landscape. Such successive transitions and diffusions are terminated when the trajectory reaches the A.M^rigor^ state. We assume that this termination is the transition from the lowest free-energy valley of the landscape of A.M^closed^ at 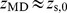 to A.M^rigor^ with the rate 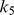. See the *Methods* for more details on the MC simulation. We should note that this MC calculation is based on the approximation that processes occurring during the transition between states, namely the lever-arm swing or chemical reactions, can be decoupled from the motions of actin and myosin within each state. This decoupling should be validated when we can assume separation of timescales among the process between states and motions within states. To evaluate the validity of this assumption, simulations of the coupled processes of transition, conformational fluctuation, and diffusive motion are necessary. A more elaborate molecular dynamics model that allows the examination of such dynamic coupling among processes is being developed [53], and we leave the application of that model to the motor problems as a future project.

**Figure 6:**
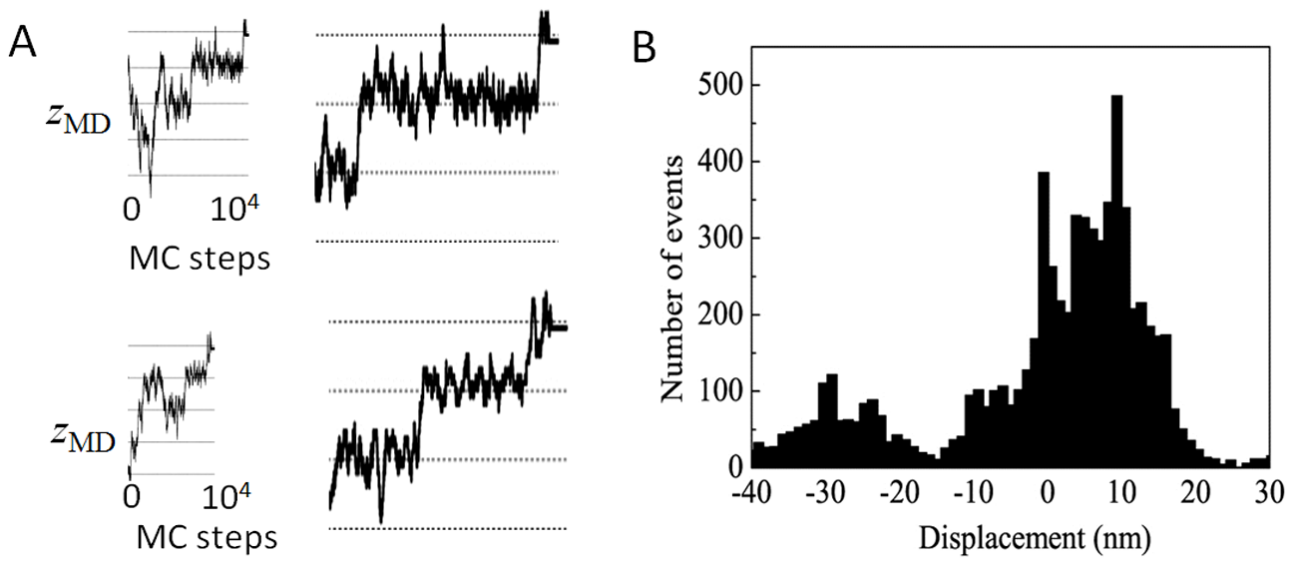
Monte Carlo simulation of a combined process of diffusion of myosin head along landscapes and transitions among landscapes. (A) Two example trajectories of myosin movement. Trajectories starting from the A.M^pre^.ADP.P_i_ state and ending in the A. M^rigor^ state (left), and their close-up from A.M^closed^ to A.M^rigor^ (right). Horizontal mesh lines are drawn every 5.5 nm. (B) The distribution of displacement of myosin head 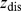 after the system reaches the A.M^closed^ state. 8,000 trajectories starting from random positions in the A.M^pre^.ADP.P_i_ state were used to calculate the distribution.

Along the MC trajectory, myosin that has begun to interact with the actin filament at an arbitrary position is attracted and weakly bound to the free-energy valley in the A.M.ADP.P_i_ state, but the position of the myosin largely fluctuates along the 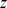-axis while it stays in the weak-binding state. After reaching the A.M^closed^.ADP or A.M^closed^ state, the myosin begins to show the biased Brownian motion. Because the closure of the 50 kDa cleft should promote the release of ADP from myosin, we assume that the lifetime of A.M^closed^.ADP is short, and thus, the persistent motion appears in the A.M^closed^ state. In the A.M^closed^ state, Brownian motion is composed of steps with a regular width of 5.5 nm, and shows both the forward and backward stepping, but is biased toward the forward direction (Fig. 6A). This biased Brownian motion is terminated when myosin reaches the rigor state.

The distribution of myosin displacement was monitored when the large positional fluctuation in the weak-binding state was reduced on the start of the displacement [11]. To compare this measurement, we monitored the simulated displacement after the system enters into the A.M^closed^ state by calculating 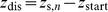, where 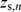 and 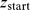 are the position of the myosin motor domain 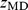 in the rigor state and that at the time when the system enters the A.M^closed^ state, respectively. 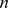 in 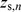 distinguishes the different strong-binding sites which are almost periodically positioned along the helical actin filament. Shown in Fig. 6B is the calculated distribution of 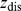, which consists of two parts, i.e., the major and minor parts. The major part is biased toward 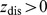 with multiple peaks separated by 5.5 nm. The major part of the distribution represents the trajectories that reach 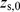. The minor part is the distribution of trajectories that reach the strong-binding site at the periodic location 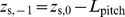.

In previous SMEs [11], [13], the displacement of S1 has been monitored at RLC near the tip of the lever arm. As will be discussed in the subsection *Contribution of the lever-arm swing*, the most probable value of the 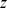-coordinate near the tip of the lever arm, 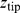, is approximately 5–7 nm greater than the 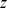-coordinate of the center of mass of myosin motor domain, 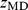, in A.M^closed^. Therefore, for comparison with the distribution of displacement of the lever-arm tip, the distribution of Fig. 6B should be shifted by several nanometers in the positive direction. With this correction, the simulated distribution of Fig. 6B reproduces the results of SME of Fig. 5 in [13]. The distribution of Fig. 6B is also consistent with the SME reported earlier [9] although the data has been differently interpreted [9] by disregarding the minor part of the observed distribution. The distributions of 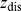 simulated with different parameterizations of kinetic rates are compared in *Fig. S2*, showing that the results are insensitive to differences in these parameters.

### Contribution of the lever-arm swing

**Figure 7:**
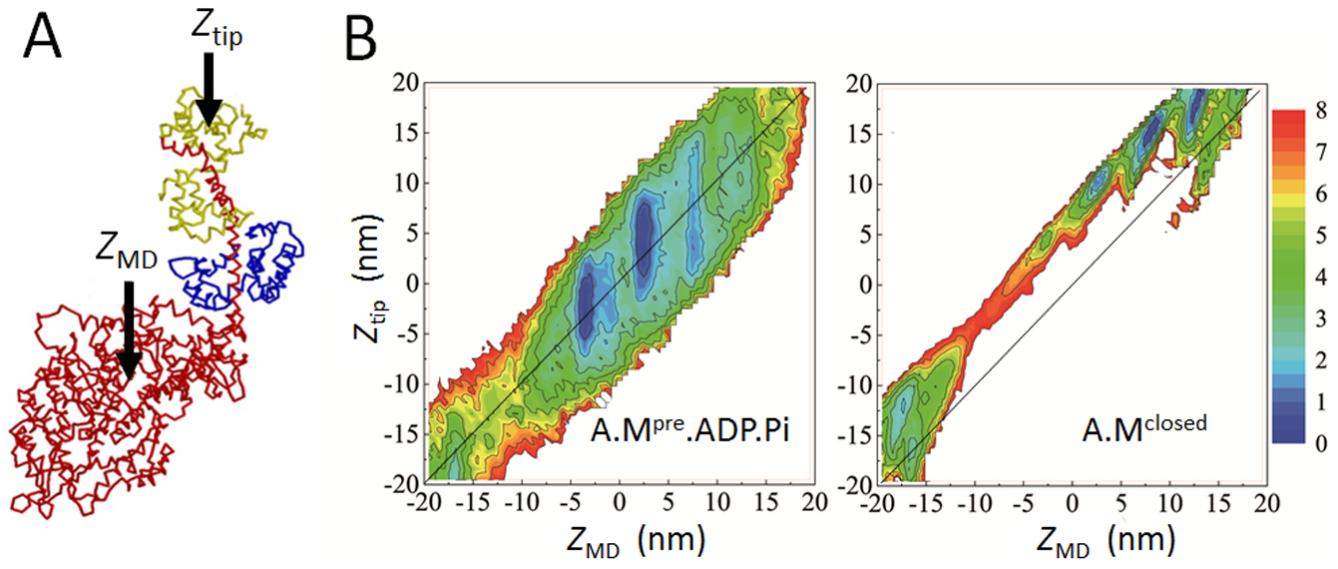
Contribution of lever-arm swing to the net displacement. (A) Position near the lever-arm tip 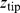 and position of the center of mass of the myosin motor domain 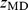 are illustrated. (B) Free-energy landscapes drawn in the plane of 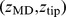 in the A.M^pre^.ADP.P_i_ state (left) and in the A.M^closed^ state (right). The diagonal line of 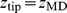 is drawn to emphasize that free-energy minima are located at 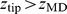.

The lever-arm swing upon the kinetic transitions in the present scheme also contributes to force generation by displacing the lever-arm tip. Fig. 7 shows the position of the center of mass of the myosin motor domain 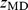 and the position in RLC near the lever-arm tip represented by 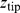 (Fig. 7A), and the free-energy landscapes drawn on the plane of 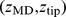 in the A.M^pre^.ADP.P_i_ state (Fig. 7B, left) and in the A.M^closed^ state (Fig. 7B, right). In A.M^pre^.ADP.P_i_ , myosin only weakly binds to actin to make the free energy insensitive to the angle of myosin to the actin surface. The free-energy basin accordingly spreads in the direction of the 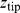 axis. In A.M^closed^, in contrast, the free-energy basin is localized at locations with 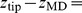
5–7 nm reflecting the post-stroke position of the lever-arm tip. From Fig. 7B, from estimated difference in location of free-energy basins in two landscapes, the net displacement of the lever-arm tip 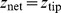 (A.M^closed^)
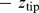
(A.M^pre^ .ADP.P_i_) is 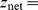
10–16 nm, in which the contribution of the biased Brownian motion 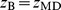 (A.M^closed^) 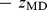 (A.M^pre^ .ADP.P_i_) is 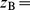
5.5–11 nm and the contribution of the lever-arm swing is 3–5 nm. The finite width of the distribution of 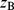 is noteworthy because of the stochastic nature of the diffusive motion, and some width of the contribution of the lever-arm swing due to the fluctuating position in the A.M^pre^ .ADP.P_i_ state should also be noted. Although the simultaneous measurement of 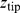 and 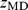 in SME has not yet been reported, it is important to acquire high resolution data for 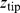 and 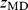 to check the validity of the discussed mechanism.

### Conformational flexibility and electrostatic interactions

As shown in the previous subsections, the biased Brownian motion of myosin arises from the global gradient of the free-energy landscape of A.M^closed^. Two crucial factors involved in this gradient are (i) the close contact of myosin and actin surfaces, which is allowed to occur only when the 50 kDa cleft of myosin is closed, and (ii) the attractive electrostatic interactions through the contact between myosin and actin [37]. In the following, we show that this contact is formed through the conformational flexibility of actin and myosin.

**Figure 8:**
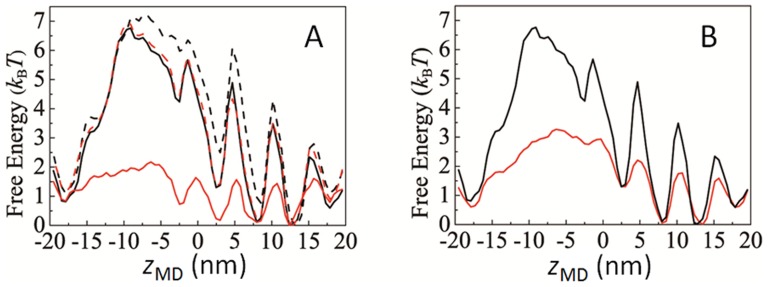
Importance of conformational flexibility and electrostatic interactions for the global gradient of the landscape. (A) Simulated free-energy landscapes in the A.M^closed^ state assuming the structurally fluctuating parts of myosin and the N-terminus of actin to be disordered without guidance of the Gō-like potentials (black solid), assuming the structurally fluctuating parts of myosin to be disordered but the N-terminus of actin to fluctuate around the estimated conformation by following the Gō-like potential (black dashed), assuming myosin to fluctuate around the estimated conformation and the N-terminus of actin to be disordered (red solid), and assuming loop 2 and loop 3 of myosin and the N-terminus of actin to be disordered with other parts fluctuating around the estimated conformation by following the Gō-like potential (red dashed). (B) Simulated free-energy landscapes in the A.M^closed^ state for 25 mM KCl solution (Debye length 1.9 nm, black line) and for 100 mM KCl solution (Debye length 0.95 nm, red line).

Some regions of heavy and light chains of myosin are structurally disordered and not determined by X-ray analysis. These disordered regions are spread over N-terminal region (residue number, 1–3), loop 1 (residue number, 205–215), loop 2 (residue number, 627–646), loop 3 (residue number, 572–574), converter (residue number, 732–737), and several regions in the ELC and RLC. In addition, the structure of the N-terminus of actin subunit is inconsistent between X-ray crystallography [54] and EM [50], indicating that this part is also disordered in solution. The importance of loop 2, loop 3, and the N-terminus of actin subunit to actin-myosin binding was shown in our previous molecular dynamics simulation [55]. In Fig. 8A four landscapes are compared with different degrees of allowed fluctuations in these regions. In one landscape, all of the disordered regions fluctuate without the guidance of the Gō-like potential, whereas in the other landscapes, the Gō-like potentials stabilizing the reference structures are assumed to regulate the fluctuation of these regions. The fluctuation of the myosin-actin surface is indispensable for the generation of the global gradient of the landscape (Fig. 8A). When loop 2 and loop 3 structures of myosin are more rigid in the simulation, the global gradient of the landscape considerably decreases, a consequence that should diminish the bias in the Brownian motion. We also find that the flexibility of the N-terminus of actin subunit enhances the biased Brownian motion; with the less flexible N-terminus of actin subunit, the barrier between the minima becomes higher, to allow the rigid N-terminus works to hinder for the diffusive motion of myosin. It would be interesting to investigate these theoretical predictions by observing the movement of myosin by following the introduction of mutations that rigidify the structure of myosin loop regions or of the N-terminus of actin.

The role of electrostatic interactions was investigated in our previous studies [37], [55] by changing the concentration of ions in solution and introducing mutations to change the charge distribution in the model. In addition, in the present simulation, the global gradient in the free-energy landscape decreases by increasing the concentration of counter ions, a result consistent with the experimentally observed decrease in the efficiency of the actomyosin motor in an *in vitro* motility assay [56] (Fig. 8B). In this study, the simulated results predict that this decrease in motor efficiency should be observed not only for the ensemble of actomyosins but also for SME.

## Discussion

Two major assumptions in the present study are that (i) myosin tends to adopt the conformations determined by X-ray and EM observations, and (ii) myosin fluctuates among these conformations as allosteric proteins fluctuate in the conformation-selection mechanism of allostery. With these assumptions, we found that free-energy landscapes for myosin movement along actin are different in the weak-binding and strong-binding states, necessarily leading to a difference in the most stable positions for myosin, which we called the weak-binding and the strong-binding sites. This difference in binding location forces myosin to move from the weak-binding to the strong-binding sites, according to the kinetic change from weak-binding to strong-binding states. We found that the free-energy landscape of this movement has a global gradient from the weak-binding to strong-binding sites; therefore, myosin shows the biased Brownian motion toward the strong-binding site as has been observed in SME. We also found that this Brownian motion is concomitant with lever-arm swing; therefore, the 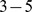 nm displacement due to the lever-arm swing and the 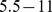 nm displacement due to the biased Brownian motion are coupled with each other to contribute to the net displacement of myosin. The simulated biased Brownian motion explains the SME data, and the theoretical results predicted that the biased Brownian motion and the underlying free-energy landscape are modified by mutagenesis to change the structural rigidity of myosin loop regions or the N-terminus of actin subunit. It is also predicted that the bias in the Brownian motion is weakened due to the increase in counter-ion concentration.

**Figure 9:**
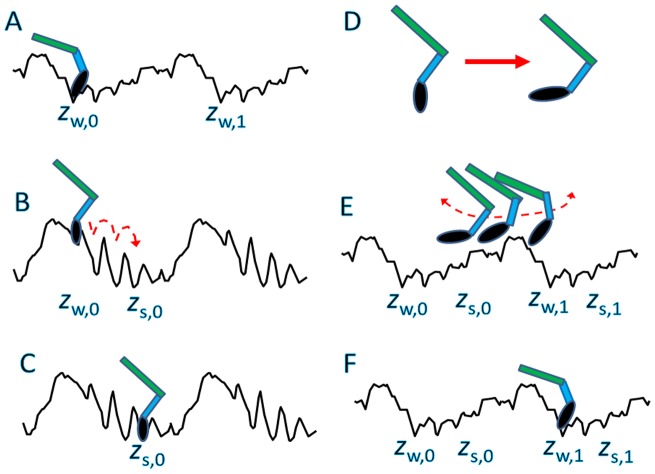
Illustration of a cycle of the suggested myosin II movement. The myosin structure is schematically represented as a composite of S2 (green) and S1, which is further composed of motor domain (black oval) and lever arm (blue). Black lines represent the free-energy landscapes for the myosin motor domain. (A) Myosin with ADP and P_i_ binds weakly to actin at around 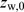. (B) After the release of ADP and P_i_, myosin begins to move along the actin filament, showing the biased Brownian motion. (C) Myosin reaches the strong binding site at 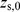 and turns into the rigor state. (D) After binding ATP, myosin detaches from the actin filament and goes through the recovery stroke, which should change the orientation of the motor domain. (E) The myosin searches for the next binding site on the actin filament through the swinging motion of S1 and S2. (F) Because of the tilted orientation of the motor domain, myosin tends to bind to the next binding site at 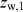. In this way, the recovery stroke shown in D plays a positive role in generating the net displacement of myosin via cycles of ATP reactions.

The displacement of S1 due to the combined effects of the biased Brownian motion and lever-arm swing arises from the dynamically changing free-energy landscape. This dynamical free-energy landscape also suggests an intriguing scenario on the mechanism by which myosin binds to actin when the S1 domain is connected to the S2 domain and further to the light meromyosin (LMM) domain. In Fig. 9, the movement of myosin with S2 in a cycle of ATP hydrolysis is illustrated. Here, one of two myosin heads is shown to emphasize the movement in an ATP cycle. Myosin weakly binds to actin in the A.M.ADP.P_i_ state at 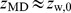 (Fig. 9A), begins to move in the 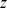 direction in the A.M^closed^ state (Fig. 9B), reaches the strong-binding site at 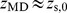, and enters the strongly bound rigor state (Fig. 9C). When myosin binds ATP, myosin detaches from actin and the myosin motor domain changes its orientation through the recovery stroke (Fig. 9D). Here, we emphasize the positive role of the recovery stroke. Because the myosin S1 is connected to S2 and LMM, the recovery stroke of the lever arm in a detached state from the actin filament does not shift the position of S2 or LMM but rather shifts the orientation of the motor domain, as illustrated in Fig. 9D. After ATP hydrolysis, the myosin begins to bind to actin. Here, we can expect that the myosin head searches for the next binding site with the swinging motion of S1 and S2 domains (Fig. 9E). With this swinging search with the motor domain oriented as in Fig. 9D, the myosin should have higher binding affinity to the binding site in the next helical pitch 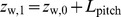 rather than to the position in the previous ATP cycle 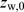 (Fig. 9F). Subsequently, after the release of P_i_ and ADP, the myosin moves toward the strong-binding site 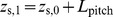. In this way, the recovery stroke should enhance the net displacement of the myosin head through the multiple cycles of ATP hydrolysis.

This suggested mechanism of myosin II movement is similar to that of processive motion of myosin V. The leading head of myosin V after ATP hydrolysis searches for a binding site to the actin filament through the swinging motion of the neck domain [17], [18]. Because the motor domain has its orientation changed by the recovery stroke in myosin V [57], the binding affinity of myosin to actin is enhanced in the forward more than that in the backward direction along the actin filament [57], [58]. The importance of the motor domain orientation on binding to the actin filament has also been suggested for myosin VI [59].

The present results showed that loop 2 of myosin is the key element in causing the biased Brownian motion. Given that loop 2 of myosin V is longer than loop 2 of myosin II, with a larger number of positive charges, the biased Brownian motion may also contribute to the processive motion of myosin V. As shown by a recent SME [19], the leading head of myosin V finds a position to bind to the actin filament through its random swinging motion, also called the Brownian search-and-catch motion. It will be interesting to investigate whether the leading head of myosin V searches for the strong-binding site through the biased Brownian motion, as discussed in the present study, at the final step of this Brownian search-and-catch process by moving along the actin filament.

The simulated result presented in this paper based on dynamical energy landscape theory is consistent with the observed structural features of myosin and actin, reproduces the SME data, predicts the effects of conformational flexibility and electrostatic interactions, and further suggests a unified mechanism for different members of myosin family. We found that the displacement of myosin head during a cycle of ATP hydrolysis is variable with respect to both the contribution of lever-arm swinging and the biased Brownian motion. These variances should be changed by the load applied to the myosin head in ways characteristic of these contributions. Such dynamically flexible responses should affect the dynamical behaviors of muscle and cardiac systems [60]. The comparisons among these system behaviors, SMEs, and *in silico* observations should open further avenues to understanding the dynamical physiological phenomena.

## Methods

### Structural models

We constructed three structural models, M^pre^, M^post^, and M^closed^ of myosin S1, each of which comprises a heavy chain, an essential light chain and a regulatory light chain. We assumed that M^pre^, M^post^, and M^closed^ are structures of myosin obtained from chicken skeletal muscle in accordance with single-molecule experiments [11], [13].

M^pre^ is the pre-stroke open-cleft structure of myosin with the bound analog of ADP and P_i_. Because the X-ray structure of myosin of chicken muscle with the analog of ADP and P_i_ is not yet available, we constructed M^pre^ using scallop myosin with ADP and VO_4_ (PDB code: 1QVI) [61] by homology modeling. Using the sequence alignment between sequences of myosin from chicken skeletal muscle and 1QVI and using the structure of 1QVI as a template, M^pre^ was computationally constructed with the software MODELLER [62]. A vanadium atom, V, was replaced with a phosphorus atom, P, to give P_i_. Parts of the myosin structure that were missing because of disorder in the template structure 1QVI were treated as flexible parts in M^pre^ fluctuating without guidance of the Gō-like potential in the model. M^post^ is the post-stroke open-cleft structure of myosin with the bound analog of ADP. M^post^ was constructed using chicken skeletal myosin without nucleotide (PDB code: 2MYS) [1], and its binding with ADP was modelled using scallop myosin with ADP (PDB code: 2OTG) [63]. We treated the missing parts in 2MYS (chicken skeletal myosin without any nucleotide) as flexible parts in M^post^ fluctuating without guidance of the Gō-like potential. M^closed^ is the post-stroke closed-cleft structure extracted from the structure determined by fitting the electron-microscope image of actomyosin complex [7]. Because M^closed^ appears in the course of relaxation from the weak to the strong actin-binding states in our simulation scheme, we assumed that structures of loops and other flexible regions of M^closed^ are not fixed as in the rigor state. We therefore treated the missing parts in 2MYS as the flexible parts in M^closed^ fluctuating without guidance of the Gō-like potential, unless otherwise noted.

We assumed that actin filament is obtained from rabbit skeletal muscle, in accordance with the single-molecule experiment [11]. A structural model of actin filament was represented as a complex of 26 subunits and was reconstructed from the X-ray structure (PDB code: 2ZWH) by positioning the adjacent subunit with 166.4 rotation and 2.759 nm translation [50]. The actin filament constructed in this way shows the 3.2 rotation for the translation of 13 subunits, amounting to 35.867 nm. We represented this structure as one exhibiting helical symmetry with approximate helical pitch 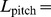
35.9 nm.

These structural models, M^pre^, M^post^, 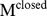, and the model of actin filament, were used as reference structures for the Gō-like potentials (see below).

### Interactions

Each polypeptide chain in the system was represented with residue-level coarse graining as a chain of connected beads of 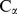 atoms. Bound ligands, Mg
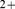
+ADP+P_i_ for the A.M.ADP.P_i_ states, Mg
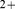
+ADP for the A.M.ADP states, were represented by all nonhydrogen atoms, whereas the nucleotide-free A.M states lacked bound ligand atoms.

The total potential energy of the actomyosin system, 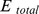, is given by
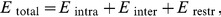
(2)where 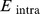 is the interaction potential within myosin (including the bound ligands) and within the actin filament, 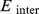 is the interaction potential between myosin and the actin filament, and 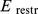 is the restraint potential on the lever-arm tip of myosin and on the subunits of the actin filament.

As shown in the following, the reference structure defined above is the minimum-energy structure in the interaction potential given by
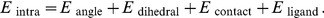
(3)The bond-angle potential 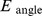 is
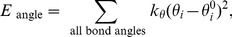
(4)where the bond angle 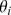 is defined as the angle formed by three successive residues 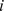, 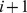, and 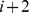, and the superscript 0 hereafter denotes the values of variables in the reference structure. The dihedral angle potential 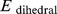 is given by
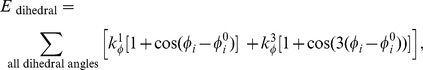
(5)where 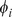 is defined as the dihedral angle formed by the four successive residues 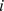, 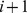, 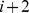, and 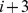. With respect to the contact interactions, all residue pairs are classified as either native or nonnative using the reference structure; for residue pair 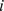 and 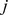 within the same chain, if at least one pair of nonhydrogen atoms are within 4.5 Å from each other in the reference structure with 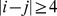, the pair 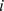 and 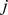 is considered a native pair. Given that there are multiple subunits within a myosin or an actin filament, residue pairs between different subunits also interact with each other by the contact potential. For the pair 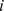 and 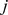 across different subunits in myosin or actin filament, if at least one pair of nonhydrogen atoms are within 4.5 Å from each other in the reference structure, the pair is considered a native pair. Otherwise, a pair within the same chain or a pair across different subunits is a nonnative pair. Contact potential 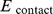 is given by
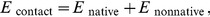
(6)and 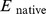 is given by
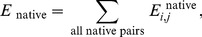
(7)where
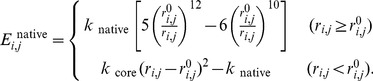
(8)

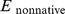
 is given by
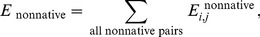
(9)where
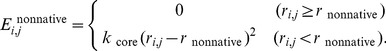
(10)The constants 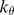, 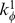, 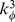, 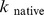 and 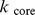 were defined as 6.67 kcal/mol/rad^2^, 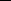
1.67
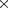
10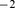 kcal/mol, 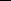
8.33
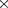
10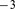 kcal/mol, 3.33
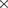
10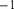 kcal/mol and 1.33 kcal/mol/Å^2^, respectively. The cutoff distance 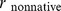 was set to be 4.0 Å.

These definitions of intramyosin or intraactin potential, including the relative strengths of bond angle, dihedral and contact potentials, are similar to those of the Gō-like model [48], [49], except for the following modifications. Bond length 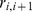 between adjacent residues along the polypeptide chain was constrained to 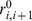 by the RATTLE algorithm [64], instead of the spring potential, to ensure the stability of the Langevin dynamics simulation. The contact potential at 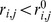 or 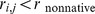 was replaced by the spring-like potential to avoid instability in the numerical integration of the Langevin equation.

Ligand contact potential 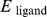 was given by the spring-like potential,
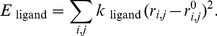
(11)The pair of ligand atoms located within 4.5 Å from each other in the reference structure interact with each other by this potential. In addition, if at least one of the atoms in the amino acid residue and an atom in the ligand are located within 4.5 Å from each other in the reference structure, that residue also interacts with the ligand atom by this potential. 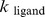 was 6.67 kcal/mol/Å^2^.

The interaction at the interface between myosin and actin, 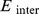, is similar to that in our previous study [37], and is composed of electrostatic and van der Waals interactions:
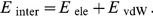
(12)The electrostatic interactions were expressed by the Debye-Hückel potential as
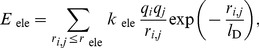
(13)where 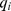 or 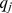 is the charge of the amino acid residue (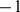 for Asp and Glu, 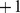 for Lys and Arg, and 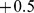 for His) or the charge of the atom in the ligand (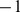 for each of the the three oxygen atoms in ADP, 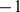 for each of three oxygen atoms in P_i_, and 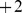 for Mg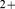). The parameters were defined as 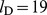 Å, 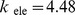 kcal  Å/mol, and 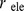 = 59.3 Å. The van der Waals interactions were given by the 12-6 type Lennard-Jones potential
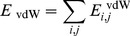
(14)with
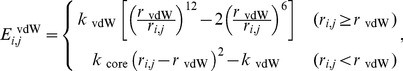
(15)where the potential at 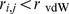 was replaced by the spring-like potential. The parameters were 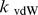 = 0.015 kcal/mol, 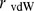 = 8.0 Å, and 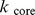 = 1.33 kcal/mol/Å^2^.

To mimic the experimental setup of the single-molecule experiment [11], we applied spatial restraints to myosin and the actin filament, respectively, as
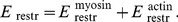
(16)The tip of the myosin lever-arm (residue number 830–843 in the heavy chain and residue number 1–83 in the regulatory light chain) was restrained with the curtain-rail potential
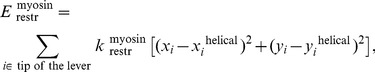
(17)where 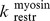 was 0.2 kcal/mol/Å^2^. The 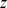-axis runs parallel to the center line of the reference structure of actin filament, and 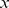 and 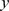 are coordinates perpendicular to the 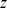-axis. We assumed that the curtain-rail runs helically around the actin filament so that the whole system has the same helical symmetry as the reference structure of the actin filament, a 3.2 rotation for each 35.867 nm. We therefore used
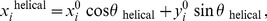
(18)

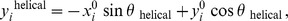
with 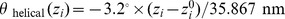. The rotation of 3.2 per 35.867 nm is so small that the helical arrangement of the curtain-rail is visually indistinguishable from the straight line along the 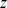-axis. However, this slightly helical arrangement aids rapid numerical convergence in WHAM by assuring the periodicity of the entire system. In accordance with the single-molecule measurement [11], no force is applied with respect to the movement of the lever-arm tip of myosin along the curtain-rail. All residues in the actin filament were restrained by the potential
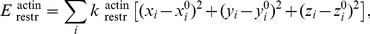
(19)where 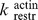 was defined as 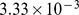 kcal/mol/Å^2^.

### Umbrella sampling by Langevin molecular dynamics

We performed the Langevin molecular dynamics simulation to sample the conformational ensemble of the actomyosin complex. The integration scheme is that of Honeycutt & Thirumalai [65], with the particle mass 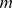 = 1.0, temperature 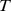 = 300 K, time step 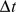 = 0.0175, and friction coefficient 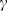 = 0.005.

We defined the two-dimensional coordinate system around the actin filament (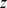, 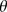), where 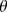 is the angle around the 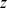-axis. The position of the center of mass of the motor domain (residue number 1–780) of the myosin head is denoted by (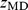, 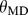). Umbrella sampling was used to enhance sampling at the high-free-energy region on the (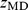, 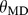) plane. The region of 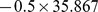 nm 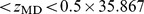 nm and 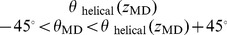 was divided into 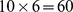 blocks, where 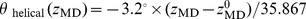 nm. We applied the umbrella potential to enhance the sampling of myosin located in each of the 60 blocks as
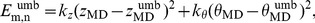
(20)where 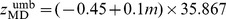 nm (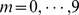) and 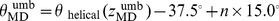 (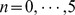). The constants 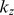 and 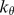 were defined as 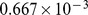 kcal/mol/Å^2^ and 10 kcal/mol/rad^2^. For each of 60 umbrella potentials, we performed 12 independent runs of Langevin dynamics for 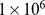 steps and the data acquired in the first half of the run were discarded. From the data obtained with each of 60 umbrella potentials, we generated the histogram of (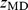, 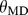) with the bin size of 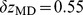 nm and 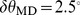. We subsequently combined these data by WHAM [51] to calculate the 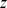-dependent free-energy landscape and the two-dimensional free-energy surface on the 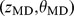 plane.

When we used only the 60 sets of the data sampled with 60 different umbrella potentials, the resulting landscape was not periodic because of the boundary effect. To avoid this numerical error, we replicated the whole data using the helical symmetry of the system as
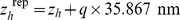
(21)

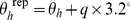
with 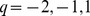, and 2, so that in total five sets of data were used. We confirmed that copying twice in both directions, resulting in five repeats of the same data, yielded a sufficiently periodic free energy landscape.

### Monte Carlo simulation

In each MCS with a time scale of 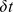, both a change in the actomyosin state and diffusion in the 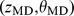 plane can occur. The procedures in one MCS were as follows: (i) the actomyosin state changed with probability 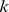 or 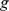 in Fig. 2, which is defined to be considerably smaller than unity. We assumed that the rates of transitions among actomyosin states are fast (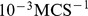) or slow (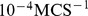) except for the transition from A.M^closed^ to A.M^rigor^, where 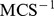 represents the inverse of an MCS. The lifetime of each actomyosin state is determined by whether the rates of approach to or departure from that state are fast or slow. Parameters were chosen to lengthen the lifetime of the A.M^pre^.ADP.Pi state: fast for 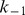, 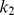, 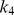, 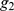, 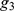 and 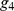, and slow for 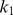, 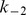, 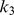, 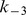, 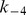 and 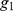. See Fig. S2 for the other choices of parameter values. (ii) If the transition to the different actomyosin state was not chosen, the diffusion of the myosin head on the two-dimensional free-energy landscape in the current actomyosin state was chosen. The trial movement of the myosin head was represented by motion along a lattice with mesh size 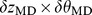. At each trial, the movement of myosin in the 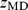 direction was chosen with probability 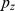, whereas movement in the 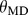 direction was chosen with probability 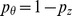 (see below for the value of 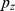). (iii) The trial move was defined by selecting either of two sites in the direction chosen in (ii) with probability 0.5. (iv) The free-energy difference 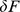 accompanying the trial move was calculated, and the trial was accepted when 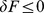 or with the probability 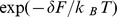 when 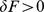, and otherwise rejected. The value of 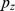 was determined as follows. One step in the 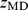 direction is the displacement of 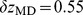 nm, whereas one step in the 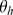 direction is the displacement of 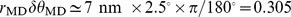 nm with 
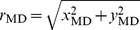
. Assuming that the Brownian motion of the myosin head is isotropic in the two-dimensional plane of 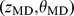, the average distance after 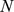 steps is 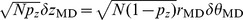, which yields 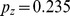.

The diffusion constant of the freely diffusing myosin head can be roughly estimated as 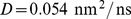 by considering the myosin head as an ellipsoid moving sidewise with semi-major axes of 8 nm and semi-minor axes of 2.5 nm in water with viscosity 0.89 pN

ns

nm at 300 K [36]. This value can be used to estimate 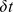, the time scale of an MCS. Because the average distance after 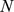 time steps is given by 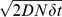, we have 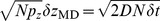, which gives 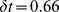 ns. Because this value is obtained by assuming the free diffusion of myosin, we should note that this estimate of 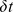 should give a lower limit. With this estimate, the trajectory of 10^4^ steps as shown in Fig. 6A has a time scale of several milliseconds or longer.

## Supporting Information

Figure S1 Two-dimensional free-energy landscapes of actin-myosin interactions for the actomyosin states defined in Fig. 2 of the main text. Because in the A.M^rigor^ state the myosin binds strongly to actin and does not show diffusive motion along the surface of the actin filament, the free-energy landscape has not been calculated for the A.M^rigor^ state, but the landscapes for the other seven states are shown.
(TIF)

Figure S2Parameter dependence of the distribution of displacement of the center of mass of the myosin motor domain in the MC simulation of successive diffusions and transitions. Starting from arbitrary positions on the actin filament in the A.M^pre^.ADP.P_i_ state, 8,000 MC trajectories of myosin movement were followed until they reached the A.M^rigor^ state. Displacement after the system enters the A.M^closed^ state was monitored. We assumed that the rates of transitions among actomyosin states are fast (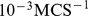) or slow (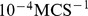) except for the transition from A.M^closed^ to A.M^rigor^, where 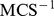 represents the inverse of a Monte Carlo step. The lifetime of each actomyosin state is determined by whether the rates of approach to or departure from that state are fast or slow. (A) Parameters are chosen to lengthen the lifetime of the A.M^pre^.ADP. P_i_ state: fast for 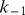, 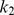, 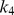, 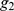, 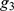 and 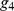, and slow for 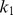, 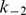, 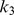, 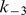, 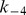 and 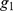. (B) Parameters are chosen to lengthen the lifetime of the A.M^pre^.ADP state: fast for 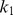, 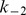, 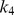, 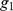, 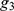 and 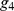, and slow for 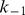, 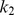, 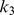, 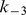, 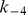 and 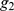. (C) Parameters are chosen to lengthen the lifetime of the A.M^post^.ADP.Pi state: fast for 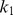, 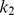, 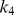, 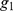, 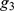 and 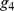, and slow for 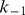, 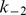, 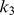, 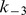, 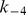 and 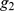. (D) Parameters are the same as in C except 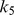 for the transition from A.M^closed^ to A.M^rigor^ states. Here, 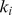 and 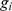 are defined in Fig. 2 of the main text. The transition from A.M^closed^ state to A.M^rigor^ state is allowed only from the valley of the lowest free energy in A.M^closed^ with the rate of 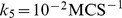 for A–C and both from the lowest valley and the 2nd lowest valley in A.M^closed^ with the rate 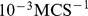 for D. A is the same as Fig. 6B in the main text.(TIF)
