# Supplementary figures and images for: Coupling of Lever Arm Swing and Biased Brownian Motion in Actomyosin
Source: PLoS Comput Biol. 2014 Apr 24;10(4):e1003552. doi: 10.1371/journal.pcbi.1003552 (PMC3998885; doi:10.1371/journal.pcbi.1003552)

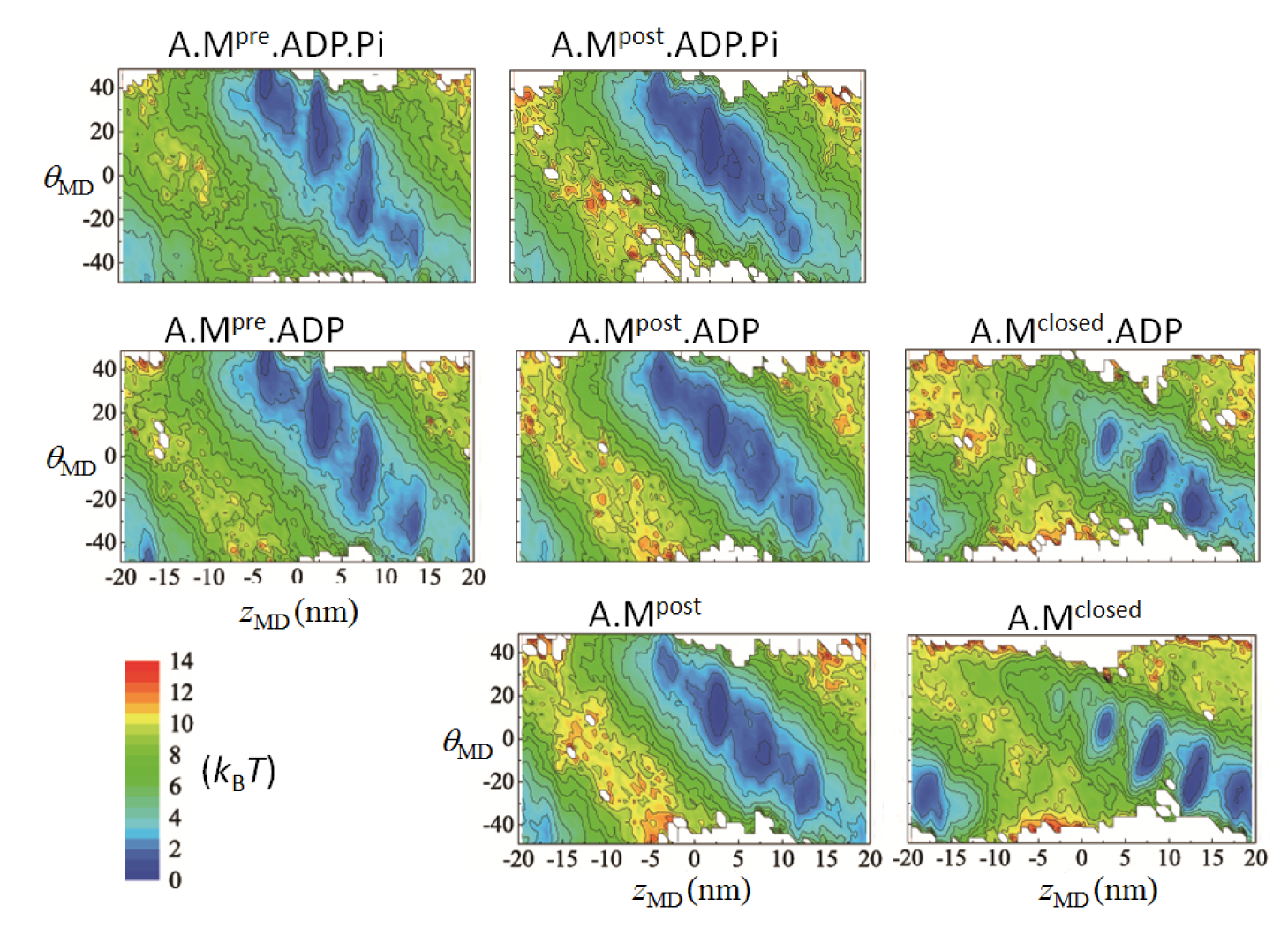

Supplement: Figure S1 — Two-dimensional free-energy landscapes of actin-myosin interactions for the actomyosin states defined in Fig. 2 of the main text. Because in the A.Mrigor state the myosin binds strongly to actin and does not show diffusive motion along the surface of the actin filament, the free-energy landscape has not been calculated for the A.Mrigor state, but the landscapes for the other seven states are shown. (TIF) [file pcbi.1003552.s001.tif]

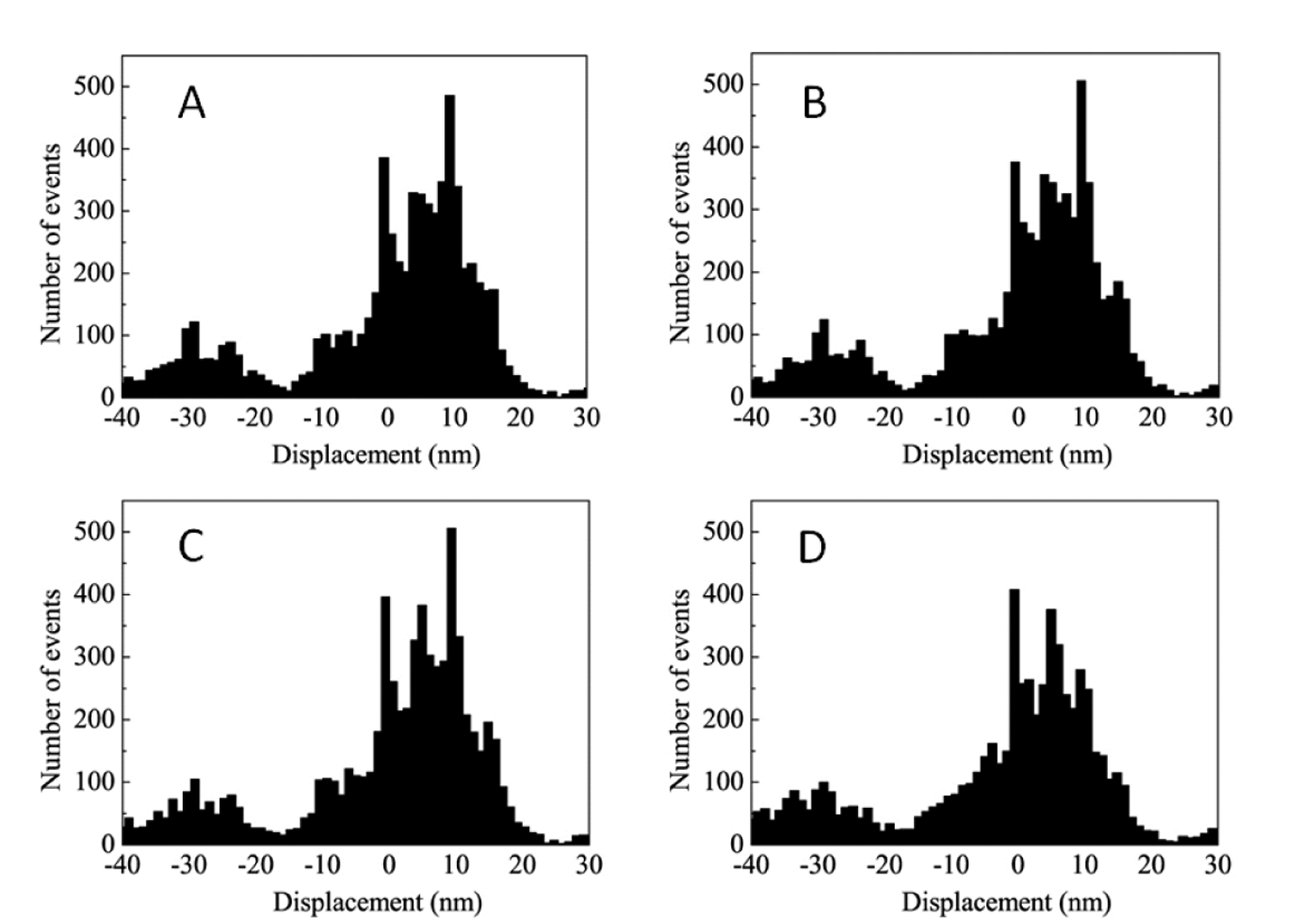

Supplement: Figure S2 — Parameter dependence of the distribution of displacement of the center of mass of the myosin motor domain in the MC simulation of successive diffusions and transitions. Starting from arbitrary positions on the actin filament in the A.Mpre.ADP.Pi state, 8,000 MC trajectories of myosin movement were followed until they reached the A.Mrigor state. Displacement after the system enters the A.Mclosed state was monitored. We assumed that the rates of transitions among actomyosin states are fast () or slow () except for the transition from A.Mclosed to A.Mrigor, where represents the inverse of a Monte Carlo step. The lifetime of each actomyosin state is determined by whether the rates of approach to or departure from that state are fast or slow. (A) Parameters are chosen to lengthen the lifetime of the A.Mpre.ADP. Pi state: fast for , , , , and , and slow for , , , , and . (B) Parameters are chosen to lengthen the lifetime of the A.Mpre.ADP state: fast for , , , , and , and slow for , , , , and . (C) Parameters are chosen to lengthen the lifetime of the A.Mpost.ADP.Pi state: fast for , , , , and , and slow for , , , , and . (D) Parameters are the same as in C except for the transition from A.Mclosed to A.Mrigor states. Here, and are defined in Fig. 2 of the main text. The transition from A.Mclosed state to A.Mrigor state is allowed only from the valley of the lowest free energy in A.Mclosed with the rate of for A–C and both from the lowest valley and the 2nd lowest valley in A.Mclosed with the rate for D. A is the same as Fig. 6B in the main text. (TIF) [file pcbi.1003552.s002.tif]
